# IL-7 and CCL19-secreting CAR-T cell therapy for tumors with positive glypican-3 or mesothelin

**DOI:** 10.1186/s13045-021-01128-9

**Published:** 2021-07-29

**Authors:** Nengzhi Pang, Jingxuan Shi, Le Qin, Aiming Chen, Yuou Tang, Hainan Yang, Yufeng Huang, Qingde Wu, Xufeng Li, Bingjia He, Tianheng Li, Baoxia Liang, Jinglin Zhang, Bihui Cao, Manting Liu, Yunfei Feng, Xiaodie Ye, Xiaopei Chen, Lu Wang, Yu Tian, Hao Li, Junping Li, Hong Hu, Jingping He, Yuling Hu, Cheng Zhi, Zhaoyang Tang, Yibo Gong, Fangting Xu, Linfeng Xu, Weijun Fan, Ming Zhao, Deji Chen, Hui Lian, Lili Yang, Peng Li, Zhenfeng Zhang

**Affiliations:** 1 Department of Radiology; Translational Medicine Center and Guangdong Provincial Education Department Key Laboratory of Nano-Immunoregulation Tumor Microenvironment, the Second Affiliated Hospital of Guangzhou Medical University, Guangzhou, Guangdong China; 2 Key Laboratory of Regenerative Biology, Guangdong Provincial Key Laboratory of Stem Cell and Regenerative Medicine, Center for Cell Regeneration and Biotherapy, Guangzhou Institutes of Biomedicine and Health, Chinese Academy of Sciences, Guangzhou, China; 3 University of Chinese Academy of Science, Beijing, 100049 China; 4 Department of Nutrition; Guangdong Provincial Key Laboratory of Food, School of Public Health, Sun Yat-Sen University, Guangzhou, Guangdong China; 5 Department of Radiology, Qianjiang Central Hospital, Qianjiang, Hubei China; 6 Department of Radiology, Shunde Chinese Medicine Hospital, The Affiliated Hospital of Traditional Chinese Medicine University of Guangzhou, Foshan, China; 7 Department of Pathology, The Second Affiliated Hospital of Guangzhou Medical University, Guangzhou, Guangdong China; 8 Guangdong Zhaotai Cell Biology Technology Ltd., Guangzhou, China; 9 Guangdong Zhaotai InVivo Biomedicine Co. Ltd., Guangzhou, China; 10 The Second Xiangya Hospital, Central South University, Changsha, China; 11 Xiangya Hospital, Central South University, Changsha, China; 12 Department of Interventional Radiology, Sun Yat-Sen Memorial Hospital, Sun Yat-Sen University, Guangzhou, China; 13 Minimally Invasive Interventional Division; Department of Medical Imaging and Interventional Radiology; State Key Laboratory of Oncology in South China, Sun Yat-Sen University Cancer Center, Guangzhou, China

**Keywords:** CAR-T cell, IL-7, CCL19, Glypican-3, Mesothelin, Pancreatic carcinoma, Hepatocellular carcinoma, Complete response

## Abstract

Although chimeric antigen receptor (CAR)-engineered T cells have shown great success in the treatment of B cell malignancies, this strategy has limited efficacy in patients with solid tumors. In mouse CAR-T cells, IL-7 and CCL19 expression have been demonstrated to improve T cell infiltration and CAR-T cell survival in mouse tumors. Therefore, in the current study, we engineered human CAR-T cells to secrete human IL-7 and CCL19 (7 × 19) and found that these 7 × 19 CAR-T cells showed enhanced capacities of expansion and migration in vitro. Furthermore, 7 × 19 CAR-T cells showed superior tumor suppression ability compared to conventional CAR-T cells in xenografts of hepatocellular carcinoma (HCC) cell lines, primary HCC tissue samples and pancreatic carcinoma (PC) cell lines. We then initiated a phase 1 clinical trial in advanced HCC/PC/ovarian carcinoma (OC) patients with glypican-3 (GPC3) or mesothelin (MSLN) expression. In a patient with advanced HCC, anti-GPC3-7 × 19 CAR-T treatment resulted in complete tumor disappearance 30 days post intratumor injection. In a patient with advanced PC, anti-MSLN-7 × 19 CAR-T treatment resulted in almost complete tumor disappearance 240 days post-intravenous infusion. Our results demonstrated that the incorporation of 7 × 19 into CAR-T cells significantly enhanced the antitumor activity against human solid tumor. *Trial registration: NCT03198546. Registered 26 June 2017, *https://clinicaltrials.gov/ct2/show/NCT03198546?term=NCT03198546&draw=2&rank=1

**Supplementary Information:**

The online version contains supplementary material available at 10.1186/s13045-021-01128-9.

## To the editor:

**Fig. 1:**
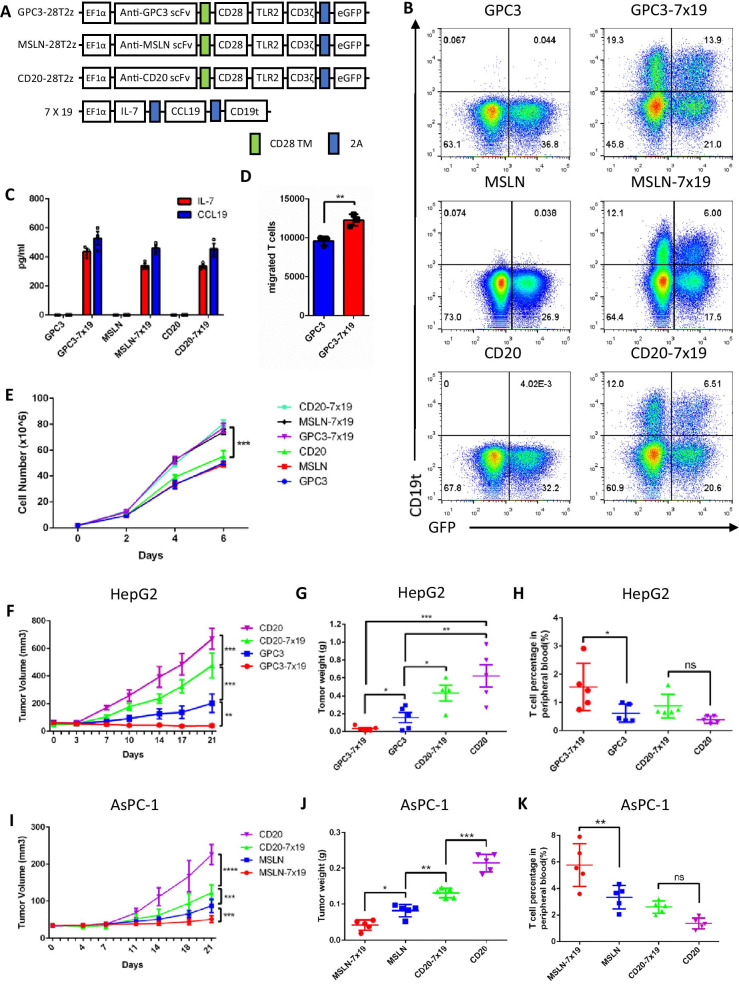
7 × 19 CAR-T cells showed enhanced antitumor activity in xenografts. **a** Schemes of a lentiviral vector of anti-GPC3 CAR, anti-MSLN CAR and anti-CD20 CAR. IL-7 and CCL19 (7 × 19) were linked through the 2A peptide, and truncated CD19 was used to indicate 7 × 19 expression. **b** The percentage of CAR and 7 × 19 expression on transduced human T cells was analyzed by flow cytometry. **c** A total of 1 × 10^6^ T cells from each group were cultured in fresh media for 24 h. The supernatant was collected, and the secretion of IL-7 and CCL19 was analyzed by ELISA. The anti-IL-7R antibody and anti-CCR7 antibody were used during culture. Open circle represents the values of individual well. Error bar denotes the S.D. **d** A total of 1 × 10^6^ anti-GPC3-7 × 19 CAR-T cells or anti-GPC3 CAR-T cells were cultured in the lower chamber of the Transwell plate for 24 h, and then the anti-GPC3 CAR-T cells labeled with CSFE were cultured in the upper chamber for 5 h. The responder T cells that migrated from the upper chamber to the lower chamber were enumerated by flow cytometry. Error bar denoted the S.D. and the results were compared with an unpaired t-test. ^*^P < 0.05, ^**^P < 0.01, ^***^P < 0.001. **e** A total of 2 × 10^6^ transduced T cells from each group were cultured in fresh media. The cell number was counted every two days. Error bar denotes the S.D. The results were compared with two-way ANOVA. ^*^P < 0.05, ^**^P < 0.01, ^***^P < 0.001. **f** Tumor volume of HepG2 subcutaneously inoculated mice. Tumor volume = (length × width^2^)/2. **g** Tumor weight of HepG2 subcutaneously inoculated mice. **h** Percentage of T cells in peripheral blood of HepG2 subcutaneously inoculated mice. **i** Tumor volume of AsPC-1 subcutaneously inoculated mice. **j** Tumor weight of AsPC-1 subcutaneously inoculated mice. **k** Percentage of T cells in peripheral blood of AsPC-1 subcutaneously inoculated mice. **f, i** Error bar denotes the S.D. The results were compared with two-way ANOVA. ^*^P < 0.05, ^**^P < 0.01, ^***^P < 0.001. **g, h, j, k** The error bar denotes the SD. The results were compared with ordinary one-way ANOVA. ^*^P < 0.05, ^**^P < 0.01, ^***^P < 0.001

A recent study showed that IL-7 and CCL19 (7 × 19) secretion promotes the infiltration and survival of murine CAR-T cells in vivo [1–3]. To further verify the effects of 7 × 19 secretion on human CAR-T cells, we constructed three CAR vectors targeting GPC3, MSLN, and CD20 (Fig. 1a) [4–6], which all contained intracellular domains of CD28 and TLR2 [7–9]. As the cargo capacity of the lentiviral vector is limited, human IL-7 and CCL19 linked through the 2A peptide were inserted into a separate lentiviral vector (Fig. 1a). We then cotransduced T cells with CAR and 7 × 19 lentiviruses (Fig. 1b). IL-7 and CCL19 were detected in the supernatant of 7 × 19 CAR-T cell cultures (Fig. 1c). 7 × 19 augmented the chemotaxis and expansion of CAR-T cells (Fig. 1d, 1e), but did not influence the cytotoxicity and cytokine secretion of CAR-T cells (Additional file 1: Fig. S1a, S1b, S1c).

We next adopted a GPC3^+^ HepG2 cell line-derived xenograft (CDX) mouse model to assess the antitumor efficacy of anti-GPC3-7 × 19 CAR-T cells. Anti-GPC3-7 × 19 CAR-T cells significantly suppressed HepG2 growth than anti-GPC3 CAR-T cells (Fig. 1f, 1g). In addition, the percentage of T cells in peripheral blood of anti-GPC3-7 × 19 group were higher than those in the anti-GPC3 group (Fig. 1h, Additional file 1: Fig. S2a). There were more infiltrated T cells in the tumors of the anti-GPC3-7 × 19 group, compared that in the anti-GPC3 group (Additional file 1: Fig. S2b). Similarly, anti-GPC3-7 × 19 CAR-T cells displayed superior antitumor efficacy in a HCC patient-derived xenograft (PDX) mouse model, compared to anti-GPC3 CAR-T cells (Additional file 1: Fig. S3). Moreover, anti-MSLN-7 × 19 CAR-T cells also displayed more potent antitumor capacity than anti-MSLN CAR-T cells in a MSLN^+^ AsPC-1 CDX mouse model (Fig. 1i, 1j, 1k, Additional file 1: Fig. S4).

Based on the preclinical results, we conducted a phase I clinical trial in advanced hepatocellular carcinoma (HCC), pancreatic carcinoma (PC) and ovarian carcinoma (OC) patients with GPC3 or MSLN expression to further explore the clinical safety and feasibility of 7 × 19 CAR-T cell therapy. The protocol of this trial is described in the supplemental materials and is depicted schematically (Additional file 1: Fig. S5, S6). Patients were enrolled based on the patient inclusion and exclusion criteria (Additional file 1: Table S1). Six patients were administered anti-GPC3/anti-MSLN-7 × 19 CAR-T cells (Additional file 1: Table S2).

**Fig. 2:**
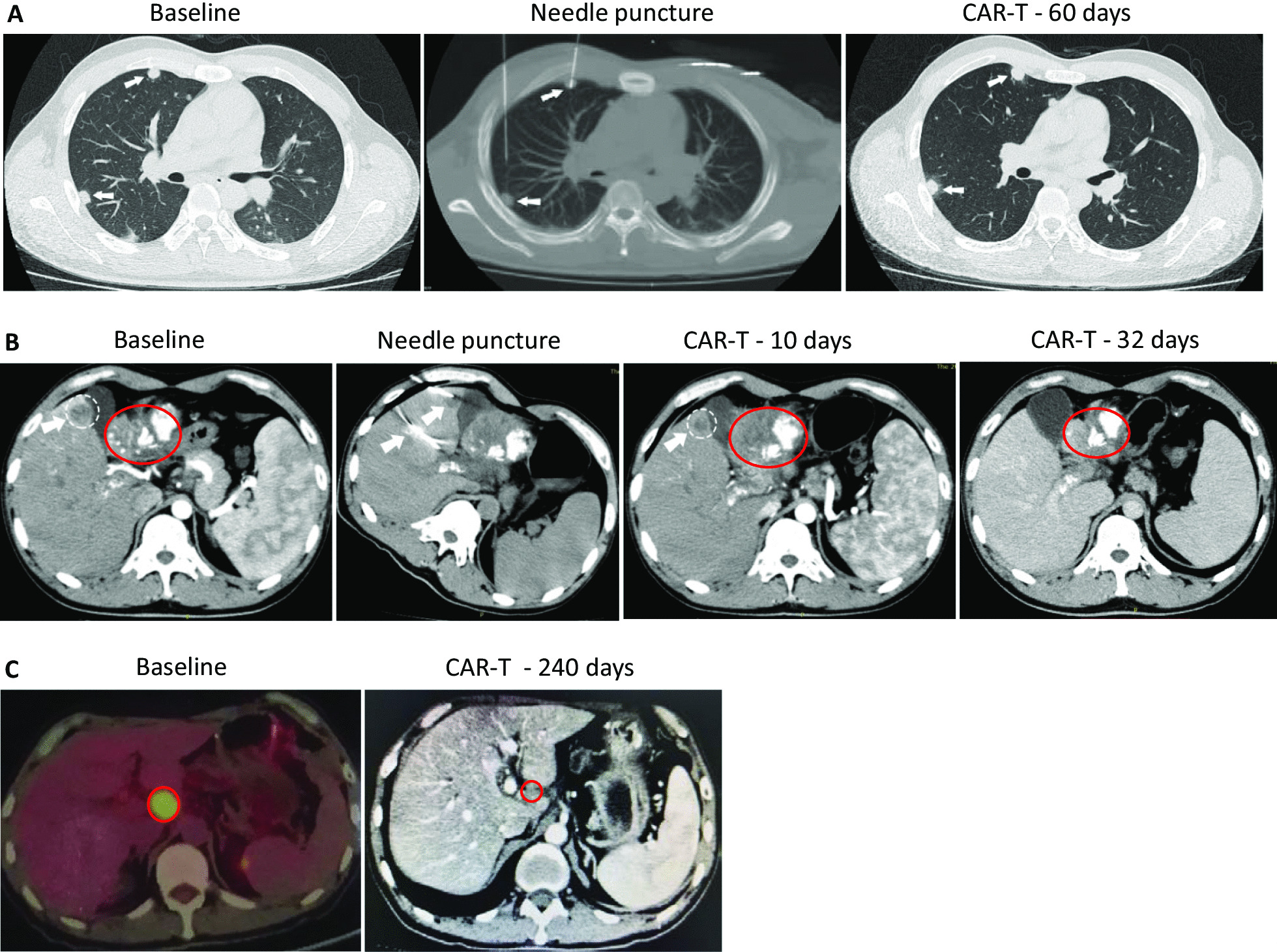
7 × 19 CAR-T cells showed remarkable antitumor activity in HCC and PC patients with GPC3 and MSLN expression. For patient GD-G/M-001, three metastatic lesions were chosen to perform fine needles CT-guided intratumor injection of CAR-T cells. **a** One nodule (0.5 cm) in the right front lung, injected with anti-GPC3 CAR-T cells as a comparison; the second nodule (0.5 cm) in the right back lung, injected with anti-GPC3-7 × 19 CAR-T cells. The results showed that the inhibited growth of anti-GPC3-7 × 19 and anti-GPC3 CAR-T cells at similar levels 60 days after injection. Tumor is indicated by white arrow. **b** For the same patient GD-G/M-001, a tumor close to the gallbladder (1.7 × 2.0 cm) was fine needle punctured under CT guidance and injected with anti-GPC3-7 × 19 CAR-T cells. 10- and 32-day follow-up CT scans showed a significant decrease in the size of the lesion and finally disappeared, respectively. At the same time, injection of the same CAR-T cells into the portal vein resulted in partial reopening of the previously obstructed portal vein. Metastatic tumor is indicated by white cycle; primary tumor is indicated by red cycle; white arrow points fine needle. **c** PET-CT and CT scans demonstrated a hepatic hilar lymph node metastasis after primary pancreatic carcinoma surgery in complete response in patient GD-G/M-005 following one intraartery and four intravein administrations of the autologous anti-MSLN-7 × 19 CAR-T cell infusion products. Tumor is indicated by red cycle

Subject GD-G/M-001 is an advanced HCC patient with GPC3 expression (Additional file 1: Fig. S7a, S7b). Three metastatic lesions were chosen to perform computed tomography (CT) guided intratumor injection of CAR-T cells. Though the sizes of two lung nodules did not change significantly 60 days after CAR-T cells injection (Fig. 2a), the liver tumor lesion (1.2 × 1.3 cm) shrunk significantly on day 10 and completely disappeared on day 32 post anti-GPC3-7 × 19 CAR-T cells injection (Fig. 2b). The patient did not suffer any toxic effects and had staging evaluation revealing partial response (PR) according to standard Response Evaluation Criteria in Solid Tumors (RECIST) version 1.1 on CT.

Subject GD-G/M-005 is an advanced PC patient with MSLN expression (Additional file 1: Fig. S7f). This subject progressed with a local lymph node metastasis (24 × 33 mm) (Fig. 2c). Anti-MSLN-7 × 19 CAR-T cells were first infused through hepatic artery with heavy fever on the night without cytokine release syndrome (CRS) or neurotoxicity [10]. Subsequently, he received intravenous infusions of the anti-MSLN-7 × 19 CAR-T cells every 1–2 months. After 5 times of the CAR-T cells infusions, CT staging revealed complete response (CR) on day 240 with the lymph node measured 8.3 × 9.6 mm and no other enlarged lymph nodes visible (Fig. 2c). He remained in normal condition.

The clinical course of the four other infused patients is shown in Additional file 1: Fig. S8 and described in Additional file 1: Table S3 and *the Materials and Methods*. Our current six-case cohort preliminary clinical study revealed no grade 2–4 adverse events or major complications; one PC patient (1/6, 16.7%) achieved CR; one HCC patient (1/6, 16.7%) achieved PR; and 2 (2/6, 33.3%) achieved steady disease (SD), demonstrating the huge therapeutic potential of 7 × 19 CAR-T cell therapy for advanced solid tumors with GPC3/MSLN expression. Due to the limited number of cases enrolled in this study, we admit this was not a comprehensive clinical trial. However, based on the safety and efficacy of 7 × 19 CAR-T cells shown by these results, we are preparing to conduct a formal phase I clinical trials of 7 × 19 CAR-T cells that specifically target liver cancer or pancreatic cancer.

## Supplementary Information

**Additional file 1.** Supplementary materials.

## Data Availability

All data are available in the manuscript or the supplementary materials.

## Abbreviations

CAR: Chimeric antigen receptor
human IL-7 and CCL19: 7 × 19
HCC: Hepatocellular carcinoma
PC: Pancreatic carcinoma
OC: Ovarian carcinoma
GPC3: Glypican-3
MSLN: Mesothelin
CR: Complete response
PR: Partial response
SD: Stable disease
PD: Progressive disease
CT: Computed tomography
RECIST: Response evaluation criteria in solid tumors
CRS: Cytokine release syndrome
