# Supplementary material for: IL-7 and CCL19-secreting CAR-T cell therapy for tumors with positive glypican-3 or mesothelin
Source: J Hematol Oncol. 2021 Jul 29;14:118. doi: 10.1186/s13045-021-01128-9 (PMC8323212; doi:10.1186/s13045-021-01128-9)
Supplement: Supplementary file 1 — Additional file 1. Supplementary materials. [file 13045_2021_1128_MOESM1_ESM.docx]

**Supplemental materials**

**Materials and Methods**

**Cell Lines**

HEK-293T cells, HepG2 cells (human liver cancer cells) and AsPC-1 cells (human pancreatic cancer cells) were purchased from ATCC. GFP- and luciferase-expressing HepG2 (HepG2-GL) and AsPC-1 cells (AsPC-1-GL) were generated by transfection of HepG2 and AsPC-1 cells with lentiviral supernatant containing luciferase-2A-GFP.

**Genes and Lentiviral Vector**

To generate CAR vectors targeting GPC3, MSLN and CD20 (as a control), the anti-GPC3 single-chain variable fragment (scFv), anti-MSLN scFv and anti-CD20 scFv were cloned into the previously reported third-generation CAR vector containing CD28, TLR2 and CD3ζ signaling domain^1-3^. Human IL-7 (ID: P13232) and CCL19 (ID: Q99731) sequence information was obtained from the UniProt database. The IL-7-2A-CCL19 sequence was synthesized by IGE Biotechnology Co., Ltd. (Guangzhou, China) and cloned into the second-generation lentiviral vector pWPXLd-2A-CD19t through Pme1 and Spe1 cloning sites.

**Lentivirus production**

Lentivirus particles were produced in HEK-293T cells via polyethyleneimine (Sigma-Aldrich, St Louis, MO, USA) transfection. The pWPXLd-based lentiviral plasmid and two packaging plasmids, psPAX2 and PMD2.G were cotransduced into HEK-293T cells in 10 cm dishes at a ratio of 3: 4: 1, with a total amount of 24 µg. Lentivirus-containing supernatant was harvested at 24, 48 and 72 h post transfection and filtered through a 0.45-μm filter.

**Isolation, Activation, Transduction and Expansion of Primary Human T Lymphocytes**

Peripheral mononuclear cells (PBMCs) were separated by density gradient centrifugation using Lymphoprep (Stem Cell Technologies, Vancouver, BC, Canada). Primary human T cells were isolated from PBMCs by negative selection using the Pan T Isolation Kit (Miltenyi Biotec, Bergish Gladbach, Germany) and activated using microbeads coating anti-CD3, anti-CD2 and anti-CD28 antibodies (Miltenyi Biotec) at a 1:1 bead: cell ratio for 48 h in RPMI-1640 supplemented with 10% FBS, 40 IU/ml IL-2, 10 mM HEPES, 2 mM glutamine and 1% penicillin/streptomycin. After activation, T cells were transduced with supernatant containing lentiviral vector expressing appropriate CAR. To generate 7×19-expressing CAR-T cells, T cells were transduced with two kinds of supernatant containing lentiviral vectors expressing CARs and 7×19 in a 1: 1 ratio. After transduction, T cells were cultured in fresh media containing IL-2 (300 IU/ml).

**Enzyme-Linked Immunosorbent Assay (ELISA)**

Enzyme-linked immunosorbent assay kits for IL-2, interferon-γ (INF-γ), TNF-α, and granulocyte-macrophage colony-stimulating factor (GM-CSF) were purchased from eBioscience (San Diego, CA, USA). Enzyme-linked immunosorbent assay kits for IL-7 and CCL19 were purchased from R&D Systems (Minneapolis, MN, USA). All ELISAs were performed according to the manufacturer’s protocols. Six days after transduction, 1×10^6^ T cells were cultured in fresh media for 24 h. The culture supernatant was collected and analyzed for the secretion of IL-7 and CCL19. The anti-CD127 antibody (clone R34-34) and anti-CCR7 antibody (clone 150503) were used during the experiment. CAR-T cells were cocultured with target cancer cells at an effector/target (E: T) ratio of 1:1 for 24 h. Then, the supernatant was collected and analyzed for the secretion of IL-2, IFN-γ, TNF-α and GM-CSF.

**In vitro tumor killing Assay**

Target cells (HepG2-GL and AsPC-1-GL) were incubated with CAR-T cells or 7×19 CAR-T cells at the indicated ratio in triplicate wells of 96-well plates. Target cell viability was monitored 24 h later by adding 100 μl/well D-luciferin (potassium salt) (YEASEN, Shanghai, China) at 150 μg/ml and measuring luciferase activity with a 450-nm laser in a luminometer. Background luminescence was negligible (<1% of the signal from wells containing only target cells). The percent viability (%) was calculated as the experimental signal/maximal signal ×100, and the percent lysis was equal to 100% viability.

**Cell Migration Assay**

The chemotaxis of the responder T cells was measured by migration through a polycarbonate filter of 5-μm poresize in 24-well Transwell chambers (Corning, Kennebunk ME, USA). Anti-GPC3 or anti-GPC3-7×19 CAR-T cells were cultured in the lower chambers. After 24 h, the responder T cells prelabeled with carboxy fluorescein diacetate and succinimide ester (CSFE) were incubated in the upper chambers for 5h. The labeled cells that migrated from the upper chamber to the lower chamber were assessed by flow cytometry.

**Flow Cytometry**

All samples were analyzed by NovocyteTM (ACEA Biosciences) or LAR Fortessa, and data were analyzed by FlowJo software. The antibodies used included anti-human CD3 (clone UCHT1), anti-human CD4 (clone OKT4), anti-human CD8 (clone OKT8), and anti-human CD19 (clone-HIB19) (Biolegend, San Diego, CA, USA). Peripheral blood, spleen and tumor tissue from xenografts were treated with red blood cell lysis buffer (Biolegend) and then stained with antibodies.

**Animal Study**

Animal experiments were performed in the Laboratory Animal Center of the Guangzhou Institutes of Biomedicine and Health (GIBH), and animal procedures were approved by the Animal Welfare Committee of GIBH. All protocols were approved by the relevant Institutional Animal Care and Use Committee (IACUC). All mice were maintained in specific pathogen-free (SPF)-grade cages and were provided autoclaved food and water.

To establish a cell line-derived xenograft (CDX) mouse model, six-week-old NOD-SCID IL-2Rγ^-/-^ (NSI) immunodeficient mice sourced from Li’s lab were subcutaneously (s.c.) injected a total of 2×10^6^ HepG2 or AsPC-1 cells into the right flanks. The progression of xenograft tumors was monitored every three days through the measurement of the length (L) and width (W) of tumors using a digital Vernier caliper, and the tumor volume (V) was calculated as V= (L×W^2^)/2. When the tumor volume reached 40 mm^3^, the mice were divided into 4 groups and received 5×10^6^ CAR-T cells suspended in 100 μl PBS intravenously (i.v.). Three weeks after T cells injection, the mice were anesthetized and then sacrificed by cervical dislocation.

To establish a patient-derived xenograft (PDX) mouse model, six-week-old NSI mice were subcutaneously (s.c.) inoculated with HCC patient tissue followed by regular protocol.

**Histological Analysis**

Tumor samples were fixed in 4% neutral formalin, embedded in paraffin, sectioned at 4-μm thickness and stained with hematoxylin, eosin and anti-human CD3 antibodies. Images were obtained on a microscope (Leica DMI16000B, Leica Microsystems, Wetzlar, Germany).

**Clinical Protocol**

A phase I clinical trial was conducted at a single center, the Second Affiliated Hospital of Guangzhou Medical University, Guangzhou, China, to verify the safety and efficiency of 7×19 CAR-T cells in human HCC, PC and OC patients with GPC3 or MSLN expression. The clinical trial was registered with ClinicalTrials.gov (NCT03198546), and all protocols and details were published online. In this study, advanced HCC, PC and OC patients were enrolled in this clinical trial and treated under this protocol. The clinical protocol received approval from the hospital ethics committee (equivalent to Institutional Review Board). Patient inclusion and exclusion criteria are shown in Table S1. Before enrollment, patients understood and signed the informed content of this clinical trial. Patients underwent biopsy to confirm GPC3 and MSLN expression in tumors by immunohistochemistry (IHC) staining before receiving CAR-T cell therapy. We collected peripheral blood mononuclear cells from patients, activated T cells and transduced them with the third-generation GPC3/MSLN-28T2z vector and the 7×19 vector to generate patient autologous anti-GPC3/anti-MSLN-7×19 CAR-T cells and anti-GPC3 CAR-T cells. After amplification and validation of the quality and killing activity of CAR-T cells, we transfused these CAR-T cells back to the patient via systemic and/or intratumor injections and closely collected the related results as needed.

**Clinical summarization of patients**

**Subject GD-G/M-001** was first diagnosed with TNM stage IV hepatocellular carcinoma (HCC, 12×13 cm in left lobe) with 9 lung metastases (4-10 mm in size) in 2018 at age 34, after more than 20 years of HBV infection and 8 years of cirrhosis. His HCC cells expressed high levels of GPC3, which was identified by IHC staining. He was first treated with 5 rounds of transarterial chemoembolization (TACE) and 3 rounds of microwave ablation (MWA) combined with lenvatinib and pembrolizumab, resulting in regression of the large liver tumor. However, a new 1.7×2.0 cm lesion appeared close to the gallbladder, and lung metastases continued to progress. He underwent steady-state T cell harvest prior to fludarabine and cyclophosphamide lymphodepleting chemotherapy before CAR-T cell infusion. GPC3-targeted CAR-T cells with or without secretion of IL-7 and CCL19 were successfully manufactured. Four intratumor injections of GPC3-7x19 CAR-T cells were performed: the tumor close to the gallbladder, cancerous thrombi in the port vein, and a nodule in the back right lung, compared with one intratumor injection of GPC3 CAR-T cells in a nodule in the front right lung on 6/15/2018 (day 1) without infusion-related complications except moderate asymptomatic pneumothorax in the right chest. He experienced no CRS or neurotoxicity. By day 10, he had a CT scan (not intended), and by day 32, he had a staging evaluation that revealed a partial response according to the mRESIST criteria. Moreover, the liver lesion close to the gallbladder disappeared, and the lung nodules with CAR-T cell injections remained the same. Unfortunately, he died from bleeding after a liver rebiopsy.

**Subject GD-G/M-002** was first diagnosed with TNM stage IV hepatocellular carcinoma (HCC, 11×12 cm in right lobe) manifested by surgery in 2018 at age 62. Her HCC cells had strong GPC3 expression by IHC. She was first treated with sorafenib with liver recurrence followed by a second surgery and multiple TACE plus radiofrequency ablation and lenvatinib. She progressed with multiple bone metastases followed by local radiotherapy and 4 cycles of nivolumab with progression. She underwent steady-state T cell harvest prior to fludarabine and cyclophosphamide lymphodepleting chemotherapy before CAR-T cell infusion. GPC3-targeted CAR-T cells with IL-7 and CCL19 secretion were successfully manufactured, and she underwent 2 intravenous infusions of CAR-T cells (3/5/2019, day 1; 3/19/2019, day 15) without infusion-related complications. She experienced slight fever and no CRS or neurotoxicity. By day 30, she had staging evaluation revealing progressive disease. She expired on hospice care and died a month late due to heart and liver function failure.

**Subject GD-G/M-003** was first diagnosed with 20 years of HBV infection and TNM stage II hepatocellular carcinoma (HCC) at age 50 in 2013. His HCC cells expressed high levels of GPC3, which was identified by IHC staining. He was first treated with 4 rounds of transarterial chemoembolization (TACE) with complete response for the next 3 years. He developed liver recurrence treated by surgical resection with continued progression, followed by lenvatinib prior to T cell harvest for CAR-T cellular immunotherapy. GPC3-targeted CAR-T cells with IL-7 and CCL19 secretion were successfully manufactured. He underwent lymphodepleting chemotherapy and an intravenous (I.V.) infusion of CAR-T cells (1x10^6^/kg) 4/12/2019 (day 1) without infusion-related complications. He experienced slight fever and fatigue without CRS or neurotoxicity. By day 28, he had staging evaluation showing stable disease by the mRESIST criteria and then received I.V. plus intra-tumor (I.T.) injection of GPC3-7x19-CAR-T cells (3x10^6^/kg, 2/3 for I.V.; 1/3 for I.T.) twice on day 29, 39. By day 60, he had staging evaluation showing stable disease with a gradual increase in serum AFP. He remained steady disease without new or ongoing study-related adverse events on day 280 after CAR-T cell infusion.

**Subject GD-G/M-004** was first diagnosed with 20 years of HBV infection, 10 years of cirrhosis, and TNM stage IV hepatocellular carcinoma (HCC, 10x12 cm in size) at age 36 in 2019. His HCC cells expressed high levels of GPC3 by IHC staining. He was first treated with 3 rounds of transarterial chemoembolization (TACE) plus lenvatinib and pembrolizumab with regression of the liver tumors. His CT scan revealed recurrence prior to T cell harvest for GPC3-7x19 CAR-T cellular immunotherapy. GPC3-7x19CAR-T cells were successfully manufactured, and then he underwent lymphodepleting chemotherapy and 2 intrahepatic artery infusions of CAR-T cells 7/8/2019 (day 1) and 8/21/2019 (day 45) without infusion-related complications. He experienced slight fever and fatigue without CRS or neurotoxicity. By day 30, he had staging evaluation showingsteady disease by the mRESIST criteria with a decrease in serum AFP. He maintained normal serum AFP and steady disease without new or ongoing study-related adverse events day 90 after CAR-T cell infusion.

**Subject GD-G/M-005** was first diagnosed with TNM stage IIB pancreatic carcinoma manifested by surgery at age 49 in October 2017. He was first treated with 6 cycles of gemcitabine and capecitabine followed by scheduled observations every 3-6 months. He progressed with a local lymph node metastasis sized 24x33 mm in PET-CT on September 2019 and was enrolled in the MSLN-7x19-CAR-T cellular immunotherapy trial. He underwent steady-state T cell harvest and cyclophosphamide lymphodepleting chemotherapy before 7x19CAR-T cell infusion. MSLN-7x19CAR-T cells were successfully manufactured and infused on 11/28/2019 (day 1) with heavy fever on the night. He experienced no CRS or neurotoxicity. He received infusions of MSLN-7x19CAR-T cells every 1-2 months and CT staging evaluations every 40-60 days until now and revealed a complete response on day 240 (07/28/2020, after 5 CAR-T infusions), with the lymph node measuring 8.3x9.6 mm (CR) and no other enlarged lymph nodes visible. He remained in normal condition without new or ongoing study-related adverse events from CAR-T cell infusion.

**Subject GD-G/M-006** was first diagnosed with TNM stage IIIC ovarian carcinoma manifested by surgery at age 40 in February 2018. She was first treated with 6 cycles of carboplatin and paclitaxel adjuvant chemotherapy. She then developed widespread recurrence throughout the abdominal cavity and received intense multiple-line chemotherapies, including doxorubicin hydrochloride liposome, nedaplatin, etoposide, gemcitabine, olaparib, apatinib, anlotinib, albumin paclitaxel, oxaliplatin, and bevacizumab, with progression prior to T cell harvest and lymphodepleting chemotherapy and MSLN-7x19 CAR-T cell infusion. MSLN-7x19CAR-T cells were successfully manufactured, and she underwent two intra-abdominal infusions of CAR-T cells 4/17/2020 (day 1) and 5/22/2020 (day 36) without infusion-related complications. She experienced no CRS or neurotoxicity. By day 38, she had staging evaluation showing progressive disease according to the mRESIST criteria and soon developed intestinal obstruction and massive ascites. She came back local hospital for palliative care.

**Statistics**

All results are presented as the mean ± SD and were analyzed via unpaired Student’s t-test (two-tailed) or one-way ANOVA. A *P* value < 0.05 was identified as statistically significant. The results were calculated, graphed and visualized by GraphPad Prism 7.0 software.

**Methods references**

1 Lai, Y. et al. Toll-like receptor 2 costimulation potentiates the antitumor efficacy of CAR T Cells. Leukemia32, 801-808, doi:10.1038/leu.2017.249 (2018).

2 Zhao, R. et al. DNAX-activating protein 10 co-stimulation enhances the anti-tumor efficacy of chimeric antigen receptor T cells. Oncoimmunology8, e1509173, doi:10.1080/2162402X.2018.1509173 (2019).

3 Lv, J. et al. Mesothelin is a target of chimeric antigen receptor T cells for treating gastric cancer. Journal of hematology & oncology12, 18, doi:10.1186/s13045-019-0704-y (2019).

**Supplemental figure legends**

**Fig. S1 Human IL-7 and CCL19 secreted by CAR-T cells did not influence the cytotoxicity of CAR-T cells.**

**(A-B)** Twenty-four-hour in vitro killing assay of 7×19 CAR-T cells and conventional CAR-T cells against HepG2-GL and AsPC-1-GL cells at the indicated E:T ratio was measured. Error bar denoted the S.D. **(C)** Cytokines secreted by 7×19 CAR-T cells and conventional CAR-T cells after coculture with HepG2 cells. Open circle represents the values of individual well. Error bars denote the S.D. The results were compared with ordinary one-way ANOVA. ^*^*P*< 0.05, ^**^*P*< 0.01, ^***^*P*< 0.001.

**Fig. S2** **Analysis of T cells in HepG2 derived CDX.**

**(A)** Percentage of T cells in peripheral blood tissue of HepG2 derived CDX. (B) Immunohistochemistry (IHC) staining of HepG2 tumors. Brown color indicates T cells infiltrated in the tumor. Scale bar is 50 μm.

**Figure. S3** **Anti-GPC3-7×19 CAR-T cells exhibited higher antitumor efficacy than anti-GPC3 CAR-T cells in a HCC PDX mouse model.**

**(A)** Tumor volume of HCC patient tumor tissue subcutaneously inoculated mice. **(B)** Tumor weight of HCC patient tumor tissue subcutaneously inoculated mice. **(C-D)** Percentage of T cells in peripheral blood tissue of HCC patient tumor subcutaneously inoculated mice. (**A**) Error bar denotes the S.D. The results were compared with two-way ANOVA. ^*^*P*< 0.05, ^**^*P*< 0.01, ^***^*P*< 0.001. (**B, C**) The error bar denotes the SD. The results were compared with ordinary one-way ANOVA. ^*^*P*< 0.05, ^**^*P*< 0.01, ^***^*P*< 0.001.

**Fig. S4 Analysis of T cells in peripheral blood of AsPC-1 derived CDX.**

**(A)** Percentage of T cells in peripheral blood tissue of AsPC-1 derived CDX. **(B)** Tumor from AsPC-1 derived CDX.

**Fig. S5 Clinical trial protocol schematic.**

The overall protocol is shown with scheduled staging procedures. ICD, informed consent document.

**Fig. S6 Consort statement/diagram.**

An overview of the number of patients screened, enrolled and infused on the protocol is shown. Of the 27 eligible patients after screening, 4 died prior to enrollment, 5 experienced disease progression that rendered them ineligible for infusion, 2 declined for other therapy and 2 withdrew consent.

**Fig. S7 Pathological detection of GPC3 and MSLN expression in the tumors of patients.**

**(A)** HE staining of biopsy tumor tissue from patient GD-G/M-001. **(B)** IHC staining of strong GPC3 expression in the HCC tissue sample of GD-G/M-001. **(C)** IHC staining of strong GPC3 expression in the HCC tissue sample of GD-G/M-002. **(D)** IHC staining of strong GPC3 expression in the HCC tissue sample of GD-G/M-003. **(E)** IHC staining of strong GPC3 expression in the HCC tissue sample of GD-G/M-004. **(F)** IHC staining of strong MSLN expression in the PC tissue sample of GD-G/M-005. **(G)** IHC staining of strong MSLN expression in the OC tissue sample of GD-G/M-006.

**Fig. S8** **CT scans of GD-G/M-002/003/004/006**

**(A)** CT scans demonstrated tumor progression in patient GD-G/M-002 following intravein administration of two autologous anti-GPC3-7×19 CAR-T cell infusion products. Tumor is indicated by red cycle. **(B)** CT scans demonstrated a tumor in steady status in patient GD-G/M-003 following three intravein and two intratumor administrations of the autologous anti-GPC3-7×19 CAR-T cell infusion product. Tumor is indicated by red cycle. **(C)** CT scans demonstrated a tumor in steady status in patient GD-G/M-004 following intraartery administration of two autologous anti-GPC3-7×19 CAR-T cell infusion products. **(D)** CT scans demonstrated ascites in progress disease in an ovarian cancer patient GD-G/M-006 following intraperitoneal administration of two autologous anti-MSLN-7×19 CAR-T cell infusion products. Ascites is indicated by red cycle.

**Table S1.** **The patient inclusion and exclusion criteria.**

| Inclusion Criteria: |
| --- |
| 1. Patients with advanced HCC, PC and OC expressing GPC3/MSLN protein |
| 2. Life expectancy >12 weeks |
| 3. Child-Pugh-Turcotte score <7 |
| 4. Adequate heart, lung, liver, kidney function |
| 5. Available autologous transduced T cells with greater than or equal to 20% expression of anti-GPC3 or anti-MSLN CAR determined by flow-cytometry and killing of GPC3^+^ or MSLN^+^ targets greater than or equal to 20% in the cytotoxicity assay |
| 6. Informed consent explained to, understood by and signed by patient/guardian. Patient/guardian given copy of informed consent. |
| Exclusion Criteria: |
| 1. Had accepted gene therapy before |
| 2. Tumor size more than 15cm |
| 3. Severe virus infection such as HBV, HCV, etc. |
| 4. Known HIV positivity |
| 5. History of liver transplantation |
| 6. Active infectious disease related to bacteria, virus, fungi, etc. |
| 7. Other severe diseases that the investigators consider not appropriate |
| 8. Pregnant or lactating women |
| 9. Systemic steroid treatment (greater than or equal to 0.5 mg prednisone equivalent/kg/day) |
| 10. Other conditions that the investigators consider not appropriate |

**Table S2. Characterizations of patients with treatment.**

| Subject ID (UPN) | Gender | Age | Histology | GPC3/MSLN score | Metastasis | Prior therapy |
| --- | --- | --- | --- | --- | --- | --- |
| GD-G/M-001 | M | 34 | HCC | +++ | Lung | 5 x TACE, 3 x MWA, lenvatinib, pembrolizumab, fludarabine, cyclophosphamide |
| GD-G/M-002 | F | 63 | HCC | +++ | bone | Sorafenib, TACE, lenvatinib,nivolumab, fludarabine, cyclophosphamide |
| GD-G/M-003 | M | 56 | HCC | +++ | None | 4 x TACE, surgery, lenbatinib fludarabine, cyclophosphamide |
| GD-G/M-004 | M | 36 | HCC | +++ | Lung | 3 x TACE, lenvatinib, pembrolizumab, fludarabine, cyclophosphamide |
| GD-G/M-005 | M | 51 | PC | +++ | lymph node | Surgery, 6 x gemcitabine and capecitabine, |
| GD-G/M-006 | F | 42 | OC | +++ | Abdominal cavity | 6x carboplatin and paclitaxel, doxorubicin hydrochloride liposome, nedaplatin, etoposide, gemcitabine, olaparib, apatinib, anlotinib, albumin paclitaxel, oxaliplatin, bevacizumab |

UPN, unique patient number; HCC, hepatocellular carcinoma; PC, pancreatic carcinoma; OC, ovarian carcinoma; TACE, transarterial chemoembolization; MWA, microwave ablation.

**Table S3. Summary of CAR-T cell treatment and clinical responses.**

| Subject ID (UPN) | Date of infusion | Route of administration | CAR^+^  (%) | Weight  (Kg) | CAR-Tdoses  (10^6^/kg) | | Toxicity | Responses | Response duration  (Months) |
| --- | --- | --- | --- | --- | --- | --- | --- | --- | --- |
|  |  |  |  |  | Doses | Total |  |  |  |
| GD-G/M-001 | 2018/06/15 | I.T. | 25.6 | 69 | 0.37 | 100 | None | PR | 2 |
|  | 2018/06/15 | I.T. | 25.6 | 69 | 0.37 | 100 |  |  |  |
|  | 2018/06/15 | I.T. | 25.6 | 69 | 0.37 | 100 |  |  |  |
|  | 2018/06/15 | I.T. | 43 | 69 | 0.62 | 100 |  |  |  |
| GD-G/M-002 | 2019/03/05 | I.V. | 21 | 48 | 1.31 | 300 | Slight fever | PD | / |
|  | 2019/03/19 | I.V. | 21 | 48 | 1.31 | 300 |  |  |  |
| GD-G/M-003 | 2019/04/12 | I.V. | 11 | 62 | 0.83 | 470 | Slight fever,  fatigue | SD | 9 |
|  | 2019/05/10 | I.V. | 30 | 62 | 1.94 | 400 |  |  |  |
|  | 2019/05/10 | I.T. | 30 | 62 | 1.35 | 280 |  |  |  |
|  | 2019/05/20 | I.V. | 30 | 62 | 2.42 | 500 |  |  |  |
|  | 2019/05/20 | I.T. | 30 | 62 | 1.21 | 250 |  |  |  |
| GD-G/M-004 | 2019/07/08 | I.A. | 21 | 73 | 1.15 | 400 | Slight fever,  fatigue | SD | 3 |
|  | 2019/08/21 | I.A. | 10.3 | 73 | 0.25 | 177 |  |  |  |
| GD-G/M-005 | 2019/11/28 | I.A. | 16 | 52 | 1.23 | 400 | Heavy fever | CR | 9 |
|  | 2020/03/25 | I.V. | 16 | 46 | 3.13 | 900 |  |  |  |
|  | 2020/03/30 | I.V. | 16.8 | 46 | 4.38 | 1200 |  |  |  |
|  | 2020/04/30 | I.V. | 16.8 | 46 | 8.77 | 2400 |  |  |  |
|  | 2020/06/19 | I.V. | 12.3 | 47 | 4.19 | 1600 |  |  |  |
|  | 2020/07/31 | I.V. | 5.6 | 49 | 0.77 | 676 |  |  |  |
|  | 2020/09/18 | I.V. | 7.7 | 49 | 2.67 | 1700 |  |  |  |
|  | 2020/10/30 | I.V. | 7.7 | 49 | 1.74 | 1110 |  |  |  |
| GD-G/M-006 | 2020/04/17 | I.P. | 10.7 | 55 | 0.97 | 500 | None | PD | / |
|  | 2020/05/22 | I.P. | 10.7 | 55 | 3.02 | 1550 |  |  |  |

I.T., intratumor;I.V., intravein;I.A., intraartery; I.P., intraperitoneal; CR, complete response; PR, partial response; SD, stable disease; PD, progressive disease.
